# Towards Risk-Based Test Protocols: Estimating the Contribution of Intensive Testing to the UK Bovine Tuberculosis Problem

**DOI:** 10.1371/journal.pone.0063961

**Published:** 2013-05-22

**Authors:** Jan van Dijk

**Affiliations:** Department of Epidemiology and Population Health, Institute of Infection and Global Health, University of Liverpool, Leahurst, Neston, Cheshire, United Kingdom; Federal University of Pelotas, Brazil

## Abstract

Eradicating disease from livestock populations involves the balancing act of removing sufficient numbers of diseased animals without removing too many healthy individuals in the process. As ever more tests for bovine tuberculosis (BTB) are carried out on the UK cattle herd, and each positive herd test triggers more testing, the question arises whether ‘false positive’ results contribute significantly to the measured BTB prevalence. Here, this question is explored using simple probabilistic models of test behaviour. When the screening test is applied to the average UK herd, the estimated proportion of test-associated false positive new outbreaks is highly sensitive to small fluctuations in screening test specificity. Estimations of this parameter should be updated as a priority. Once outbreaks have been confirmed in screening-test positive herds, the following rounds of intensive testing with more sensitive, albeit less specific, tests are highly likely to remove large numbers of false positive animals from herds. Despite this, it is unlikely that significantly more truly infected animals are removed. BTB test protocols should become based on quantified risk in order to prevent the needless slaughter of large numbers of healthy animals.

## Introduction

**Figure 1:**
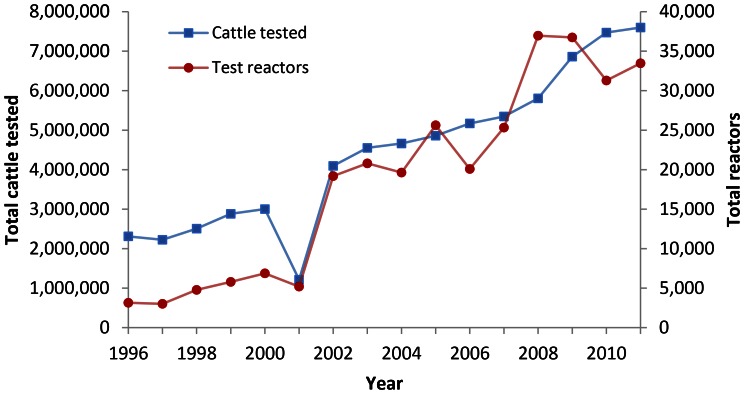
BTB testing in Great Britain, 1996–2011 [14]. The total number of cattle tested for the presence of an immune response to *Mycobacterium bovis*, and the total number of cattle testing positive in these tests, are shown.

Bovine tuberculosis (BTB), caused by *Mycobacterium bovis*, is an important notifiable disease of cattle with, in countries where milk is not routinely pasteurised, serious zoonotic potential [1]. In the United Kingdom, since the introduction of regular testing of the national herd in the 1950s, clinical BTB is very rarely witnessed. However, the compulsory eradication programme has become an ever-increasing financial burden. For example, between 1998 and 2009, annual UK government spending on BTB rose from £25 million to £108 million with, in 2009, testing and compensation paid to farmers making up 85% of the costs [2]. Against a background of a declining UK cattle population, both the total number of BTB tests carried out and the number of test reactors removed are increasing year on year (figure 1).These substantial efforts towards disease transmission control apparently not achieving their target, some have argued for the abandonment of UK BTB regulation, for lack of cost-effectiveness [3]. Costs to the cattle export industry associated with adopting such a policy [4] are likely to be much lower than current spending on disease eradication [3].

The total number of test reactors removed from the UK herd appears to be well predicted by the number of tests conducted (figure 1). Traditionally, this has encouraged workers to intensify test regimes in a bid to, ultimately, remove the last reactor from the national herd. Whether this is possible depends first on the sensitivity of our tests and second on the rate at which cattle become infected. Recent work has indeed focussed on the sensitivity of BTB testing [5], [6] and suggested that the disease cannot be eradicated through testing alone if re-infection of herds cannot be controlled [7]. However, it appears that a second important question, whether high-frequency testing actually represents the *optimum* strategy, also needs to be addressed. Apart from the soaring test-associated costs, if an increasing number of animals is tested more frequently then, inevitably, more ‘false positives’ (FPs) are identified. These represent compensation costs, considerable distress to farmers and the risk of introducing other diseases into the herd when replacement animals are purchased. From an epidemiological and economical point of view, the removal of any extra FP may only be justified if this is offset by the benefits of removing more true positives in the process. In this respect, test specificity is key. If animals in a herd, previously free of disease, test FP, the knock-on effects can be dramatic: the testing frequency in this herd is increased until it once again appears free of BTB while the testing frequency of other farms in the parish is also influenced. In other words, there is a feedback mechanism where the finding of a reactor triggers more testing which in turn may identify more reactors: test reactors drive the number of tests conducted (figure 1). As suboptimal test specificity could make the (perceived) BTB problem spiral out of control, it is important that the test-associated reactor identification rate is analysed. The fact that test outcomes cannot be verified by a ‘gold standard’ test adds urgency to such an analysis. It can be very difficult to reach the BTB diagnosis even after very thorough *post mortem* examinations of cattle [8], and absence of the bacterium is impossible to prove. BTB tests do not detect the presence of *M. bovis* itself but of an immune response to it, i.e. historic infection which may or may not still be present. It is widely thought that animals, once infected, will remain so for the rest of their life but robust data on the proportion of animals clearing the bacterium is lacking.

The single intradermal comparative cervical tuberculin test (SICCT) is the established ‘first line’ test used on all farms. This test is thought to be very highly specific but this is traded off with suboptimal sensitivity [9], [10]. When it identifies a test reactor, and subsequent post-mortem and/or lab tests cannot confirm the presence of *M. bovis*, the herd in question undergoes 60-day short-interval SICCT re-testing, until it tests clear [11]. If the initial test reactor is found to be infected however, the herd is retested until two clear tests are achieved, making use of the ‘severe interpretation SICCT’. The latter improves test sensitivity [6] at the expense of specificity, the value of which is not widely published. In 2006, mainly for problem herds, where repeated testing keeps identifying reactors, the gamma interferon test (γIFN) was formally added to the GB BTB test regime. This test is also more sensitive but less specific than SICCT [12], [13]. In 99% of applications, it is used as an additional (parallel) test to SICCT, with the aim of improving overall test sensitivity. Over the period 2007–2011, between 22 and 30 thousand bovines were tested in this manner per annum [14]. Results of parallel testing shows that γIFN, in some herds, may identify eight times as many positive animals as SICCT [12]. When these are slaughtered, on average, the presence of *M. bovis* can be confirmed in fewer than one in five animals [12], [15], prompting intense discussion on the true infection status of the non-confirmed cows [16].

Sensitivity and specificity do not only vary between tests but also when a test is applied to different populations [17]. For BTB tests, their values may depend on the proportion of infected animals in a stage of infection detectable by the test, whether tested animals have been in contact with infected cattle [18], infection with mycobacteria other than *M. bovis* [9], [19] and several non-TB status related factors, such as cross-reactivity between SICCT and γIFN [20], the storage of blood samples and other laboratory-associated influences [12] and co-infection with macro parasites [21]. Therefore, when a BTB tester turns up at a farm gate, the exact sensitivity and specificity of the test to be carried out that day are unknown. However, the literature provides us with estimates and so confidence limits for both can be established.

**Figure 2:**
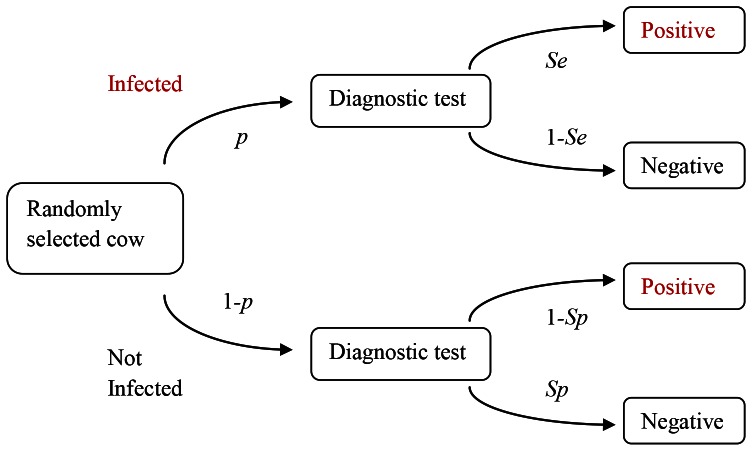
The four possible outcomes of testing a randomly selected animal for the presence of infection [21]. *p* = prevalence of infection in the herd, *Se* and *Sp* = sensitivity and specificity of the test, respectively. The positive predictive value (PPV) equals the probability of the animal being truly positive (*p* * *Se*) divided by the total probability of a positive test result ((*p* * *Se*)+(1-*p*) * (1-*Sp*)).

Sensitivity and specificity give us the probability that an animal, given its infection status, tests positive or negative. However, in the field, epidemiologists are confronted with a test result and the probabilities that an animal that has tested positive is truly infected or that an animal that has tested negative is truly free of disease, have to be calculated. These probabilities are known as the ‘positive predictive value’ (PPV) and ‘negative predictive value (NPV), respectively. Both depend not only on the sensitivity and specificity of a test but also on the prevalence of disease tested population (figure 2). It is possible to adjust sensitivity and specificity of tests to maximise PPV or NPV in specific test situations. The ‘severe interpretation’ SICCT is an example of this. In search of optimum test protocols, test behaviour might have to be adapted in a more fluent manner, for example targeted towards estimated herd prevalence levels.

Applying basic epidemiological principles and probability theory, this paper explores the likely contribution of UK BTB testing itself to the increasing prevalence. First, the probabilities of both available tests giving FP, and false negative (FN), results is quantified at the individual animal level. Subsequently, these tests are applied to all cows in herds of various sizes and the test-associated total number of expected FPs is predicted. SICCT and γIFN are compared against each other in terms of the likelihood of removing more true positives, and suggestions for improvements to the current test regime are made.

## Methods

### Probability of Individual Animals Testing False Negative and False Positive

The probability of a randomly chosen animal falsely testing negative or positive was modelled as a function of test sensitivity, test specificity and herd prevalence as follows [22]. The probability of the animal being infected despite testing negative, which equals 1– negative predictive value (NPV), is the probability that the animal has TB yet tests negative divided by the total probability of the test being negative (figure 2):
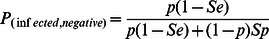
(1)with *p* = within-herd prevalence, *Se* = test sensitivity, *Sp* = test specificity. Similarly, the probability of the animal testing being free of disease despite testing positive, which equals 1-PPV, is the probability that it is not infected yet tests positive, divided by the total probability of the test being positive (figure 2):
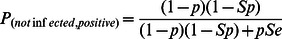
(2)
*Se* and *Sp* were modelled stochastically. For the standard interpretation of SICCT, and γIFN, at least 11 field-derived estimates of these variables were identified in the literature: *SICCT Se*: 0.898 [10], 0.914 [23], 0.72 [24], 0.955 [25], 0.75 [26], 0.824 [27], 0.73 [28], 0.551 [29], 0.909 [30], 0.909 [31], 0.80 [32], 0.75 [33], 0.935 [33] (Mean 0.8188, SD 0.1165); SICCT Sp: 0.963 [13], 0.999 [23], 0.98 [24], 0.978 [25], 1 [31], 0.999 [34], 1 [35], 0.98 [36], 1 [37], 0.94 [38], 0.968 [39] (Mean 0.9825, SD N/A); *γIFN Se*: 0.816 [28], 0.955 [31], 0.85 [32], 0.843 [35], 0.94 [38], 0.818 [40], 0.937 [41], 0.73 [42], 0.877 [43], 0.849 [44], 1 [45], 0.882 [46] (Mean 0.8748, SD 0.0740) and *γIFN Sp*: 0.958 [13], 0.994 [28], 0.877 [31], 0.93 [32], 0.926 [33], 0.888 [37], 0.94 [38], 0.98 [39], 0.991 [40], 0.94 [45], 0.92 [46], 0.90 [47] (Mean 0.9370, SD 0.0369). The distribution of estimates of sensitivity and specificity of γIFN, and of sensitivity of the skin test, did not differ significantly from a normal distribution (Kolmogorov-Smirnov test statistic ≥0.133, df ≥10, p≥0.11) and were modelled as such. The distribution of test specificity for SICCT was negatively skewed and modelled as a triangular distribution with as minimum value 0.94 and as both likely and maximum value the mode, 1.0. A Monte-Carlo simulation (in this and in all following simulations 10,000 iterations were run) of the behaviour of this triangular distribution projected a mean of 0.980 (95% CI 0.953–0.998). All computer simulations were undertaken using a random number generator (PopTools, CSIRO, Australia) within an Excel spreadsheet (Microsoft Inc., USA). To study the behaviour of the two tests with regards to individual animals, a Monte-Carlo analysis was performed for both models and tests, at ‘true’ prevalence levels between 0 and 1, increasing prevalence at increments of 0.1.

### Numbers of Cows Testing False Negative or False Positive at the Herd Level

First, probability densities of the number of cows predicted to test FP when SICCT and γIFN are applied to a zero-prevalence herd, containing the UK average of 117 dairy cows [48], were drawn. The probability of a cow testing FP (Pc) was modelled as 1-*Sp* and probability densities, both based on the mean and 95% CI literature- derived values and DEFRA-published estimates of *Sp* (0.999 for SICCT [10], [23], [49] and 0.967 for γIFN [13], [50]) drawn as the binomial distribution of the number of animals in the herd and the associated probability of a single animal testing FP (B(*n*, 117, Pc); with *n* the number of FPs). Similarly, the effect of herd size (*H*) on the probabilities of finding *n* = 1–3 FP animals in the screening test (*Sp* = 0.999) was modelled as B(*n*, *H*, Pc) in 50-cow steps (50 to 600 cows). The effect of small changes in *Sp* on the probability of multiple cows (*n* = 2–3) testing FP was explored using an alternative UK BTB data-derived *Sp* estimate of 0.9967 [18].

Second, test performance was assessed in three field situations. The results of herd tests are normally not disclosed to the public. However, at least three high-profile test results, amongst which farms that appealed to the High Court to order a re-test, have been put in the public domain [16], [51], [52]. Regardless of whether test results on these farms are representative or not, it is informative to calculate the degree to which they can be explained by the performance of the tests used. On all three farms, SICCT was carried out alongside γIFN. Based on the test results, which are summarised in table 1, three estimates of TB prevalence in these herds were made as follows. The first estimate was based on the number of animals confirmed to have TB at slaughter. For farm East Sussex, a single animal identified as TB positive in the slaughterhouse, just prior to the test, was used for this estimate. Second, an estimate was based on the number of animals testing FN in the skin test. This was achieved by solving equation (1) for *p*, giving
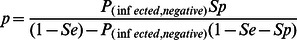
(3)


**Table 1:** Test results of three herds where SICCT and γIFN test were carried out in parallel [16], [51], [52].

Farm	Yewdall	Higher Burrow	East Sussex
Cattle tested	450	430	600
SICCT positive	1	14	4
γIFN positive	89	86	107
Confirmed*	5	16	0**

* Test-positive cows revealing BTB lesions post mortem/positive culture of *M. bovis*.

** One culled cow had been identified as positive at slaughter, prior to the test being ordered.

**Figure 3:**
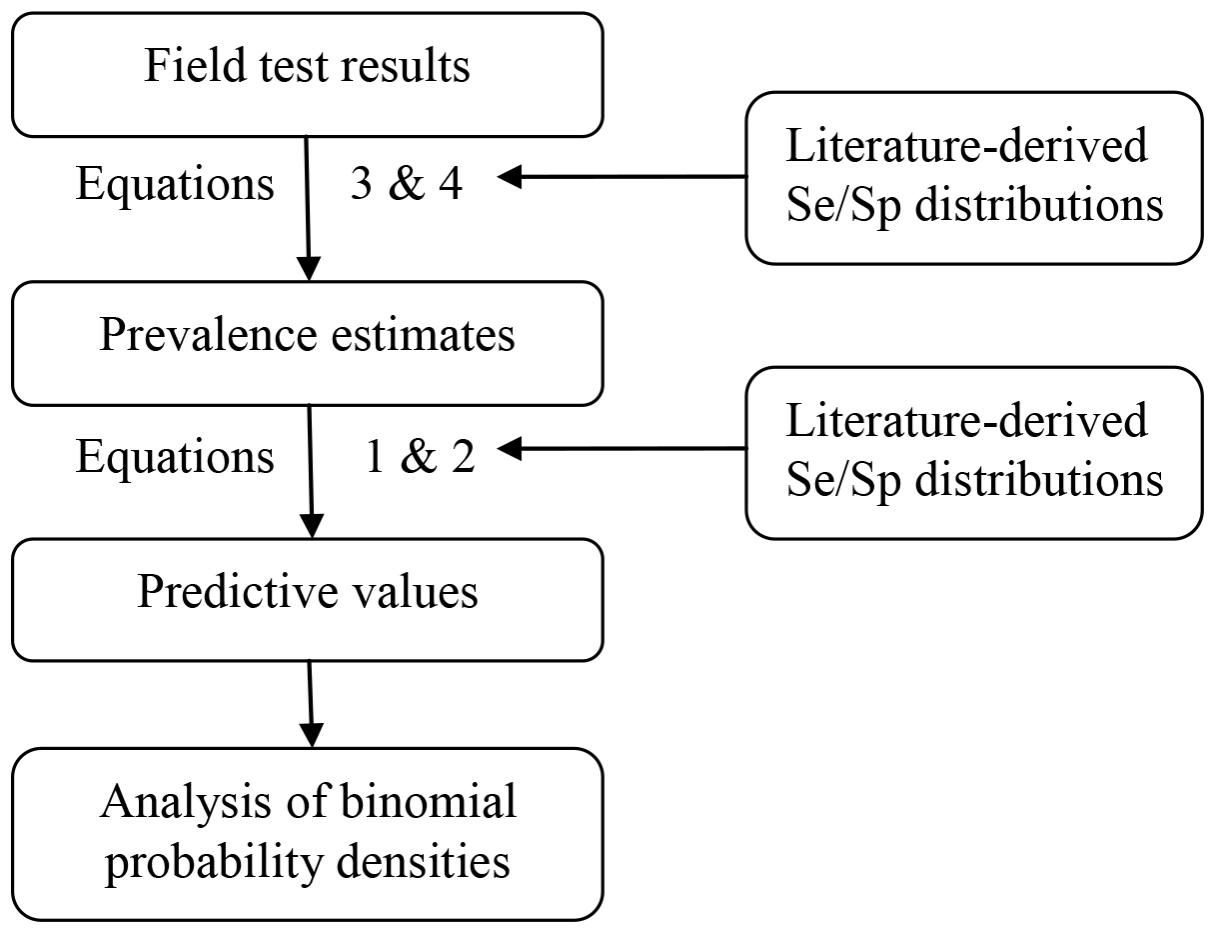
Algorithm of analyses performed on data from three example herds.

The mean prevalence was estimated by Monte Carlo simulation, using the *Se* and *Sp* densities given above. Third, an estimate was made assuming that all positive γIFN tests had correctly identified TB-infected animals. In this case, the prevalence is simply calculated as
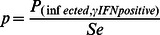
(4)and once again estimated by Monte Carlo simulation. In order to evaluate the behaviour of the tests at very high prevalence levels, the artificial prevalence level of 0.5 was also modelled. All thus achieved prevalence estimates were modelled in equations (1) and (2) and Monte Carlo analyses run, once again making use of the *Se* and *Sp* densities described above. Subsequently, the calculated mean probabilities of falsely identifying an animal from these herds as BTB negative or positive were used to assess differences between the two tests at the herd level. To this extent, the probability density of the predicted number of FN and FP animals identified by the tests carried out in the three herds, was modelled as the binomial distribution of the number of animals identified as negative/positive by the test and the associated probability of FNs/FPs at the estimated prevalence level. For example, for Higher Burrow farm, for the estimated prevalence level of 0.0723, the number of FN animals left in the herd was modelled as B(n = 416, P = 0.0145) for the skin test and B(n = 344, P = 0.0104) for γIFN. Differences between the number of false negatives left in the herd by both tests, and false positives removed, were tested for significance assessing the degree of overlap of the two probability densities as follows. For the assessment of FN left in the herd, 10,000 samples of both probability densities were taken randomly by the computer. Assuming that the skin test would leave more false negatives in the herd, for each sampling, the value for the skin test was deducted from that for γIFN; the P-value for differences between tests, representing the overlap between the two binomial distributions of the probability densities, was assessed as the number of times the outcome of deducting the two values gave a negative result, divided by 10,000. For the assessment of differences between the number of false positives removed from the herd, the same procedure was followed but the γIFN value was deducted from the skin test value. The workflow described above is summarised in figure 3.

Last, as probability density estimates described above had been based on values for individual animals within relatively large herds, with regards to FPs, the potential influence of herd size on the performance of both tests was investigated for herds containing 50 to 250 cattle. At simulated prevalence levels of 0.05, 0.1, 0.2 and 0.3, the probability of identifying FP animals was calculated as described above. For both tests, the predicted number of FP animals were modelled as binomial probability densities, increasing the number of cattle in 50-animal steps. Differences in the predicted number of FP animals identified by both tests were, at each prevalence and herd level, tested for significance through Monte Carlo sampling as above.

## Results

### Probability of Individual Animals Testing False Negative and False Positive

**Figure 4:**
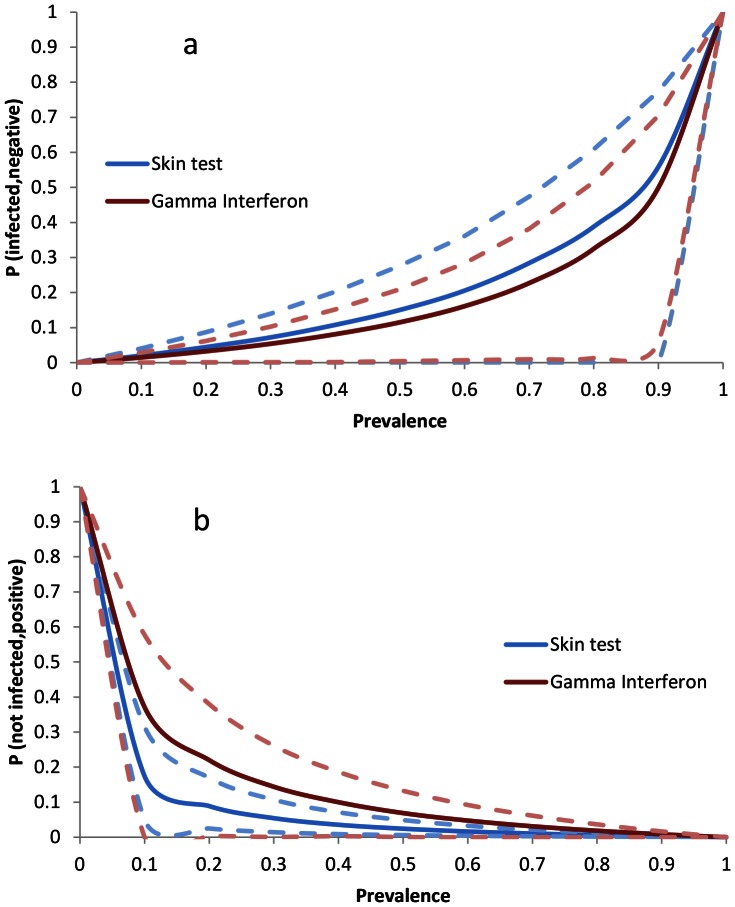
The probability of false SICCT and γIFN results in individual animals. The probabilities of a randomly chosen animal which has tested negative to be infected (1-NPV; figure a) and an animal which has tested positive to be uninfected (1-PPV; figure b) are shown as a function of TB prevalence in the herd. Dotted lines give the 95% confidence limits.

For the two tests, γIFN and SICCT (standard interpretation), the mean probabilities of a randomly chosen animal that has tested negative in reality being infected (1-NPV) run very close together at each level of BTB herd prevalence (figure 4a). At low prevalence levels, the predicted probability for FNs is low, and confidence limits are both narrow and overlap for both tests. Only above a true prevalence level of approximately 0.3 (i.e. approximately one in three animals infected in a herd), the probability of a false-negative test increases more rapidly for both tests and the 95% confidence limits for become very wide. The mean probabilities of animals testing positive in reality being free of disease (1-PPV) declines very rapidly especially between prevalence levels 0 and 0.1 (figure 4b). For SICCT, the mean probability declines more rapidly and remains lower than γIFN at all prevalence levels. The upper confidence limit of SICCT is below the mean value of γIFN but the lower 95% CI for the latter completely overlap with those of SICCT, making differences between the tests not statistically analysable at this level.

### Numbers of Cows Testing False Negative or False Positive at the Herd Level

**Figure 5:**
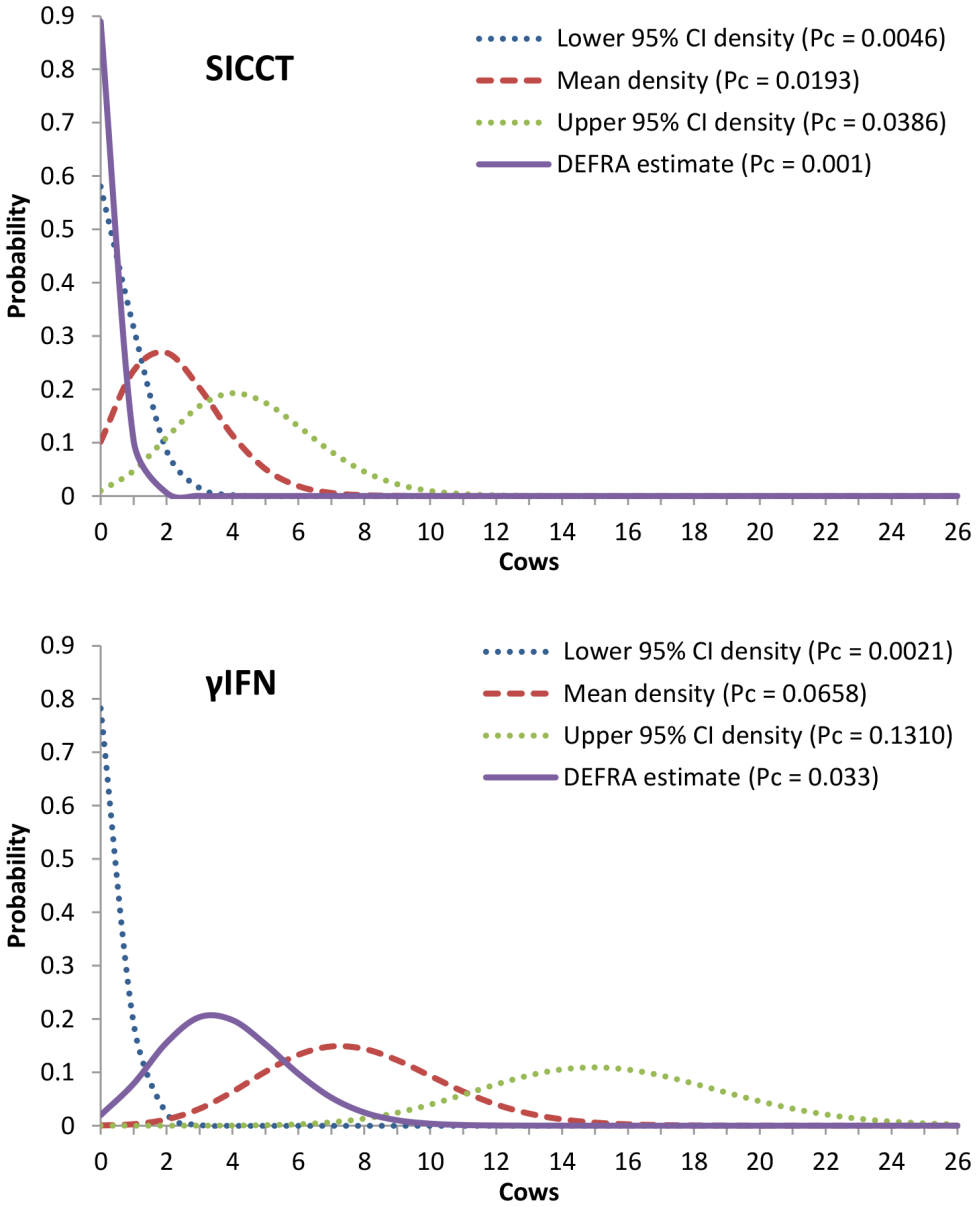
The number of cows predicted to test false positive in an average (117-cow) herd. Probability densities drawn for SICCT and γIFN with the probability of individual cows testing positive (Pc) equalling 1-*Sp*. The red probability density gives the animals based on the average values of *Sp* taken from the literature (95% confidence-limit densities also given), the purple density is based on the DEFRA-published *Sp* estimate (standard interpretation for SICCT).

**Figure 6:**
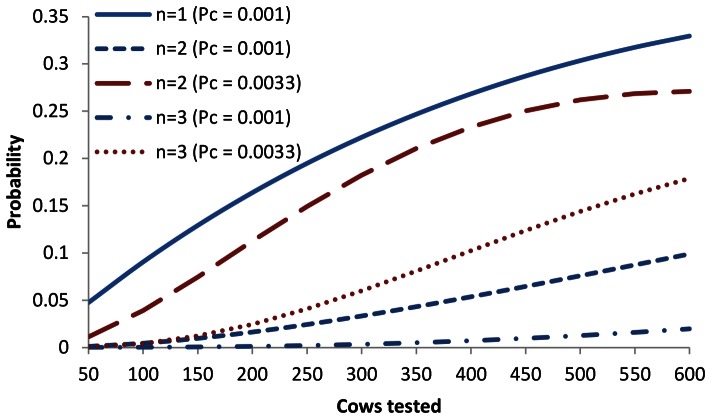
The probability of testing *n* cows SICCT false positive as a function of herd size. The DEFRA *Sp* estimate of 0.999 (Pc = 0.001) was used; for n = 2–3, the 0.9967 *Sp* estimate by Hartnack and Torgerson [18] (Pc = 0.0033) was also applied.

In a UK-average size, zero-prevalence herd, based on the mean of published *Sp* values, 9 out of 10 herd tests would be predicted to identify 1 or more FPs; the most likely value is 2 cows per test (figure 5; NB: to improve visual clarity, these and following binomial probability densities were drawn as ‘continuous’). The DEFRA estimate of 0.999 is above the upper 95% CI for published *Sp* and is predicted to yield a single FP in one out of ten herd tests, the probability of identifying 2 or more FPs being virtually zero. Average published estimates of γIFN *Sp* correspond with between 2 and14 FPs each test (figure 5). The DEFRA estimate is within the upper 95% CI and gives a probability of 0.98 to identify between 1 and 8 FPs, the most likely number being 3 cows. With respect to the influence of herd size (figure 6), at the DEFRA-estimated SICCT *Sp* level, the probability of finding a single FP increases from approximately 1 in 20 tests (*H* = 50) to 1 in 3 tests (*H* = 600). In herds containing 600 cows, 1 in 10 tests would be expected to identify 2 FPs while the probability of finding 3 or more FPs is less than 0.02. However, when *Sp* drops slightly to 0.9967 [18], there is a probability of 0.27 (one in 3.7 herd tests) of identifying two FPs and a probability of 0.18 (1 in 5.6 herd tests) of three FPs.

**Figure 7:**
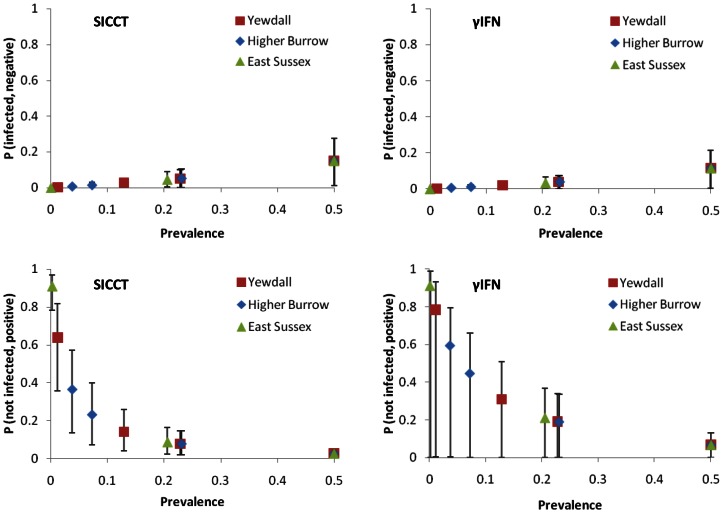
The probability of false BTB test outcomes in individual animals on three example farms. Using SICCT and γIFN tests, the mean probability of a randomly chosen animal which has tested negative to be infected (1-NPV; top figures) or an animal which has tested positive to be uninfected (1-PPV; bottom figures) is shown at various calculated TB prevalence levels. The herd prevalence levels were calculated from the actual test results at farms ‘Yewdall’, ‘Higher Burrow’ and ‘East Sussex’. Error bars represent 95% confidence limits.

**Figure 8:**
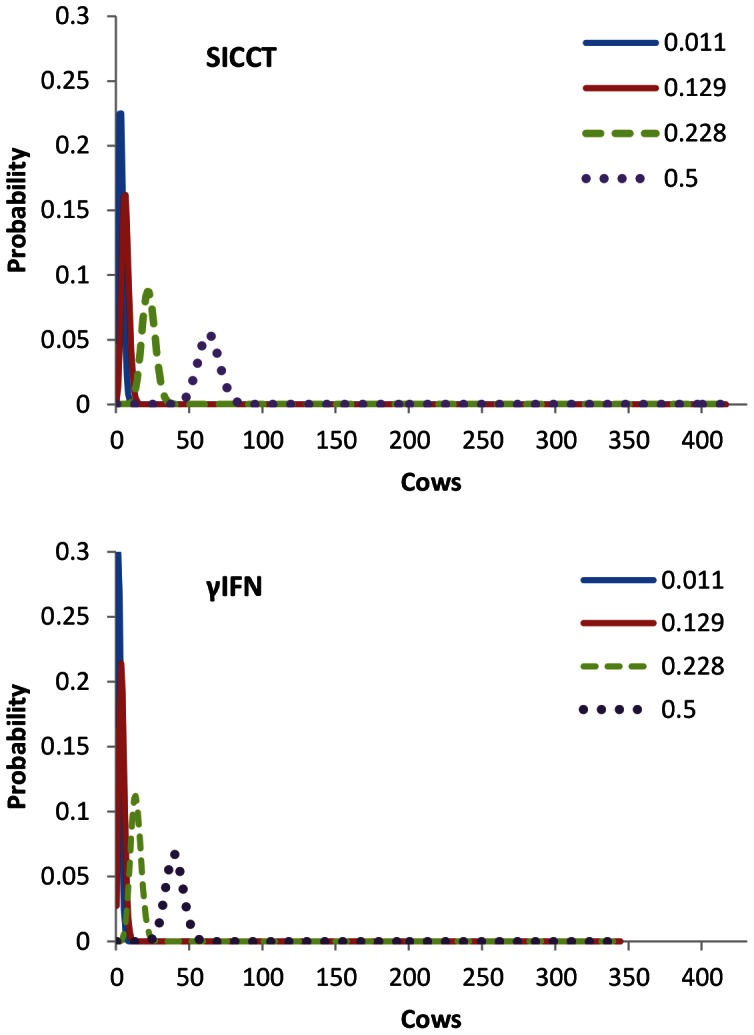
The number of infected cows predicted to have tested negative at Higher Burrow farm. Probability densities are shown with the different colours representing various calculated prevalence levels, the value of which is given. Probability densities at prevalence levels 0.011 and 0.129 overlap. γIFN identified 72 more animals as positives and therefore the herd was reduced by this number before constructing the probability densities.

**Figure 9:**
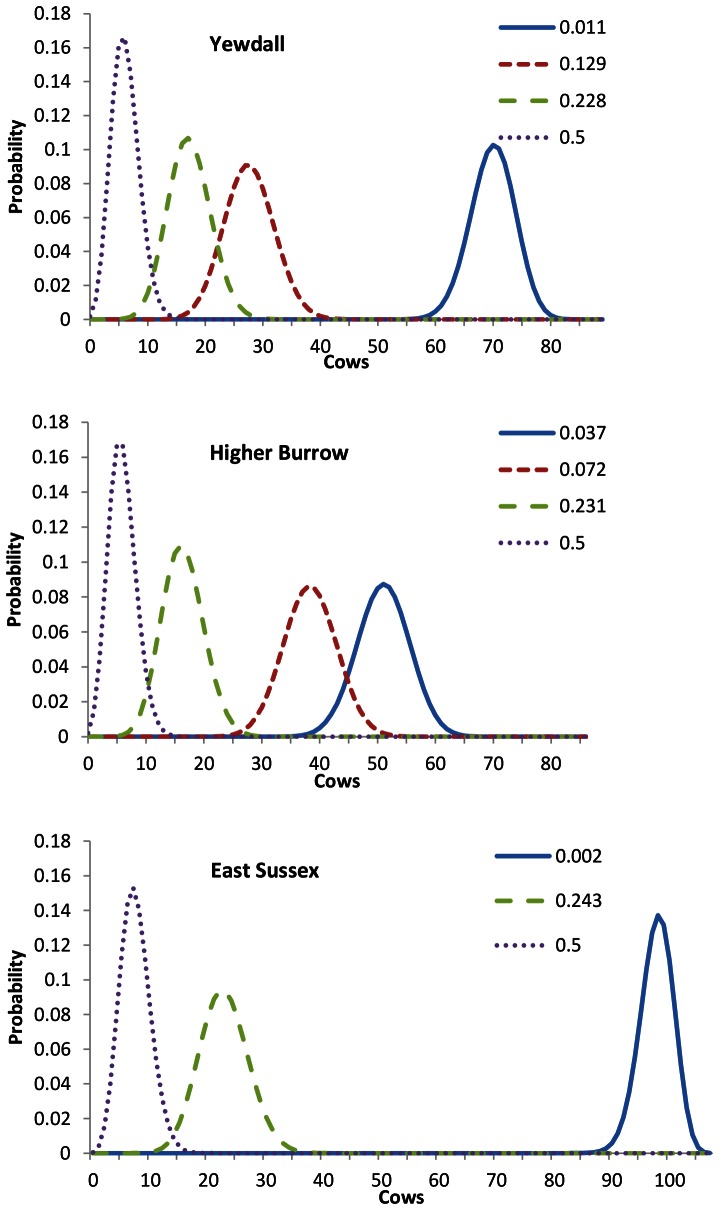
The number non-infected cows predicted to have tested γIFN positive in three herds. The mean binomial probability densities are shown for farms ‘Yewdall’, ‘Higher Burrow’ and ‘East Sussex’. Different coloured lines represent various calculated prevalence estimates, the values of which are given.

**Figure 10:**
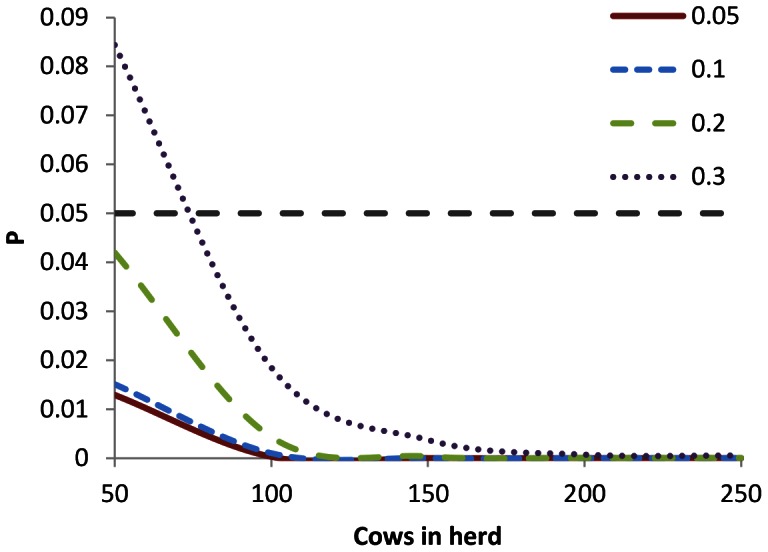
Herd size and the predicted difference in numbers of γIFN and SICCT false positives. The probability of a null hypothesis stating that γIFN and SICCT give an equal amount of false positives being true is given for herds containing 50–250 cows. The coloured lines represent different levels of BTB prevalence in the herd, the value of which is given; the dotted line gives the α = 0.05 significance level. Only in very small herds, at high prevalence levels, is SICCT projected not to significantly outperform γIFN.

For the three real farm test situations, ‘Yewdall’, ‘Higher Burrow’ and ‘East Sussex’, the 1-NPV and 1-PPV probabilities for individual animals attached to the prevalence estimates are given in figure 7. With regards to 1-NPV, especially at the lowest prevalence levels, the tests produce virtually identical results. However, mean probabilities for 1-PPV are higher for γIFN at all levels. At all prevalence levels, γIFN-lower confidence limits also touch the zero line, suggesting that this test may behave more unpredictably than the SICCT. When these probabilities are subsequently applied at the herd level, the pattern of the probability densities relating to FNs are very similar; they are illustrated for Higher Burrow farm in figure 8. It appears that, even when the tests are repeated many times when applied to large herds of 430–600 cows, the mean performance of both tests with respect to FNs cannot be distinguished. Relating P-values for differences between the two tests in terms of the probability densities of the total number of FNs and FPs are given in table 2. At prevalence levels up to approximately 0.2, γIFN is not predicted to remove significantly more infected animals from the herd than SICCT (P≥0.10). However, at prevalence levels of around 0.2 (i.e. one in five animals in the herd infected) and above, γIFN performs significantly better (P≤0.05). The probability densities for the predicted number of animals to have been falsely identified as positive by γIFN in the three herd tests is given in figure 9. At lower prevalence estimates, a very large proportion (0.65–0.95) of the slaughtered animals is predicted to consist of healthy animals. At the high prevalence level indicated by γIFN in tests carried out in these herds, approximately one quarter of the ‘positive’ animals is predicted to be truly BTB negative, while even at an unrealistically high prevalence level of 0.5, up to 15 animals are predicted to test false positive. At each prevalence level, the number of false positives identified by γIFN is predicted to be significantly (P≤0.005) higher than that identified by the skin test (table 1). Herd size (i.e. the number of tests carried out) does appear to affect these predictions very little (figure 10). Only in small herds (*H* = 50), at very high prevalence levels (between 0.2 and 0.3), does γIFN not identify significantly (P≤0.05) more false positives than the skin test.

**Table 2:** P-values for differences in the probability densities of total numbers of false negative and false positive cattle in SICCT and γIFN expected at various prevalence estimates (given in figure 4).

Prevalence estimate	On post mortem/culture	On skin test false negatives	On γIFN results	0.5
*False negative*				
Yewdall	0.207	0.1007	0.046	0.0027
Higher Burrow	0.2171	0.169	0.0533	0.0061
East Sussex	0.1255	N/A	0.0489	0.0013
*False positive*				
Yewdall	<0.0001	<0.0001	<0.0001	<0.0001
Higher Burrow	<0.0001	<0.0001	<0.0001	0.0005
East Sussex	<0.0001	N/A	<0.0001	<0.0001

## Discussion

Over the period 1998–2010, in herds, previously clear of BTB, testing one or more animals SICCT positive, the presence of *M. bovis* could consistently be confirmed in just over half of these ‘breakdowns’ [14]. At the individual animal level, 50–80% of animals testing SICCT positive neither show typical BTB lesions at post-mortem examination nor the growth of *M. bovis* in culture media [9]. It is therefore important to know whether these herds/individual animals were in fact 1) in a stage of infection not detectable by post-mortem or lab tests or 2) free of disease after clearing an historic infection or 3) wrongly identified as positive by the test. A recent latent class analysis by Hartnack and Torgerson [18] showed bacterial culture sensitivity to be as high as 0.98 and SICCT specificity to be substantially reduced, to 0.65, in animals that have been in contact with infected cattle. This suggests that SICCT positive, culture negative animals may indeed have cleared an infection. Currently, only the test-associated proportion of animals testing false positive can be estimated. In this respect, it needs to be remembered that even highly specific tests have the potential to identify false positive animals, especially when they are repeated many times, during whole-herd tests. Using the DEFRA estimate of SICCT specificity, in an average BTB-negative herd of 117 cattle, just over one in ten herd tests would be predicted to wrongly identify a single cow as positive. It would therefore appear that a relatively small proportion of positive herds suffer solely test-associated breakdowns. From Karolemeas *et al.* [53] it can be deducted that, over the period 2003–2006, 13,170 herd breakdowns were recorded of which 39.5% (*n* = 5,196) remained unconfirmed. 59% of these, i.e. 23% of all breakdowns, concerned single animals testing positive; at a specificity of 99.9%, nearly half of these (*n* = 1,372) are estimated to be false positive herd tests. The proportion of herds predicted to test false positive is highly sensitive to small specificity fluctuations. For example, if the average literature-derived SICCT specificity estimate of 0.98 is applied to the same 117-cow herd, it is predicted that only one in ten BTB-free herds tests negative (figure 5). This does not reflect field experience with the test, and the true average *Sp* value may exceed 0.9967 [18]. However, it is clear that, in order to be able to estimate the true contribution of ‘test error’ to new BTB herd breakdowns, it is crucial to calculate robust specificity estimates. Especially in large herds, a drop in *Sp* as small as 0.0023 leads to a large increase in the probability of finding multiple FPs (figure 6). As test specificity is co-determined by the epidemiology of other mycobacteria [9], [19], it may change over time and estimates should be re-evaluated regularly. The 0.999 estimate appears based on a crude calculation [10] and a study carried out in 1975 [23]. Traditionally, estimating test specificity was problematic, as it involved testing disease-free herds, but Bayesian statistical methodology is now well established [54]. Even at a *Sp* of 0.999, the calculated probabilities of finding one or more FPs during screening tests (figure 6) are cause for concern, especially given the current UK trend of increasing herd size. ‘Breakdown’ herds will subsequently only be declared ‘disease free’ after standard SICCT 60-day interval tests have been carried out until a single negative herd test result is obtained, at a significant costs to farmer and tax payer. It may not be appropriate to expect zero positive test outcomes for large herds where the presence of *M. bovis* cannot subsequently be confirmed. Such herds may still eventually turn out to be infected, the likelihood of confirmation of which depending on disease progression within the 60 days. But the likely benefit of the second, and subsequent, tests can be estimated from the number of reactors in the first test: if the prevalence is indicated to be low then, for the subsequent test, 1-PPV is very high (figure 4) and any animals removed from the herd likely to have tested false positive. If the herd tested false positive as a result of cross-reaction with other bacterial infections, it may continue to test positive for several tests.

If BTB is confirmed to be present in animals identified as positive in the screening test, the ‘severe interpretation’ SICCT is applied at 60-day intervals, until two consecutive clear herd tests are achieved. This ‘severe interpretation’ SICCT was recently estimated to increase the relative test sensitivity from 0.81 to 0.85 [6]. However, inevitably, this will be traded off with a loss of test specificity; as outlined above, even small losses would make it highly likely the herd tests positive again. Only one estimate of severe interpretation SICCT specificity (0.888 [24]) could be obtained from the literature and therefore the performance of this test could not be analysed in detail. However, the likely consequences of sacrificing test specificity for sensitivity can be gleaned from the analysis of the behaviour of the γIFN test, which is largely applied parallel to SICCT after several short-interval tests fail to produce negative herd test results; at this moment in time, BTB prevalence is likely to be low and γIFN behaviour unpredictable (figures 4 & 7). If tests carried out have already removed the truly infected animals, then, in the average herd, γIFN is predicted to nearly always wrongly identify some animals as positive (figure 5). But also in herds were infection is still present, γIFN is predicted to identify large numbers of false positive animals and significantly (P<0.0001) more so than SICCT. For the three large herds for which test data was available, at the lowest prevalence estimates, between 65 and 95% of animals identified as positive by γIFN were predicted to have been false positives. Even at the high prevalence estimates indicated by the γIFN test itself, it would be predicted that 10–30 false positive animals would removed. Meanwhile, each testing round would induce the next. Published data indeed show that a disproportionally large number of reactors is revealed at short interval tests [10]. 30% of breakdowns continue for more than 240 days, i.e. require 4 or more follow-up tests before a herd is declared BTB free again, the strongest predictor of the number of re-tests being whether the breakdown was confirmed or not [53]. Costs associated with a 60-day follow-up test interval may be justifiable if they are balanced with significant prevention of within-herd BTB transmission. Although temporally explicit estimates are lacking, cattle-to-cattle transmission rates would appear to be low [10], [55], [56]. If the main route of infection is wildlife-to-cattle then frequent retesting of breakdown herds is unlikely to be cost-effective.

In search of the optimum test strategy, it can be argued that removing healthy animals from a herd is the price to pay for removing more truly infected animals and thereby eradicating BTB more rapidly. However, this analysis showed that γIFN, despite removing significantly more animals from a herd, at realistic prevalence levels, is unlikely to remove significantly more truly infected animals. Intuitively, it may appeal to apply a more sensitive test to chronic ‘problem populations’, to remove infected animals more rapidly. But the present analysis shows that more sensitive, less specific, tests may only be beneficial in high prevalence populations instead. Published BTB confirmation rates on γIFN- positive animals, based on post-mortem examination and culture of *M. bovis* from carcasses, appear to reflect as much; they run at 67.7% in high-prevalence herds were whole-herd slaughter is considered but 17.4% in herds where BTB is detected for the first time [15]. Since its introduction in 2006, the large number of positive animals identified by γIFN has been explained in terms of the test expressing such sensitivity towards early infections that it diagnoses these correctly without visible lesions being found [12], [13] and this has become the established veterinary hypothesis. However, this analysis shows that observations made in the field can be explained by the test characteristics alone. Given the wide range of field-derived estimates of test characteristics, and the trade-off between sensitivity and specificity itself not having been quantified, all possible combinations of both parameters were drawn from distributions. Consequently, confidence intervals on model output were very wide and this is why differences in probabilities could not be statistically separated at the level of the individual animal. γIFN confidence limits were always wider than SICCT, reflecting the field-experience that its results are highly unpredictable.

γIFN was developed as a complementary test to the skin test, to 1) increase overall test sensitivity in herds with a continuing high incidence of TB through the parallel use of both tests followed by removal of all positive animals, and 2) increase overall specificity in herds where a large number of animals show non-specific reactions to the skin test by removing only animals positive in both tests [9]. Its use remains advocated especially for ‘parallel testing’ [15]. It is well established that γIFN and SICCT do not identify the same animals as positive [12]. As the tests show no signs of conditional dependence [54], the probability of animals being truly infected when testing positive in both tests is high [57]. However, the assumption that, where two tests are positive albeit in different animals within one herd, both tests must be right is questionable. A recent robust Bayesian analysis of overall performance of the combined use of both tests estimated overall specificity to be only 85% [54]. Sacrificing, on average, 15 ‘false positive’ cows out of every 100 clearly can only be economically justified if not only significantly more true positive animals are removed but also the (long-term) costs of leaving these in the herd outweighs the costs of removal of healthy animals in this test and the subsequent test it triggers.

The apparent increase in overall UK BTB prevalence, in the face of an ever-increasing test-and-cull intensity, has largely been ascribed to the presence of a wildlife reservoir. Recently, it was proposed that a dampening of immune responses through co-infection with endemic helminth parasites may contribute to a failure to detect BTB where it is present [21]. The present study initiates the estimation of the direct contribution of BTB testing itself. Frequent TB testing, year on year, may also contribute to our problem in a different way. As immune responses are measured, it follows that, paradoxically, it will be the cattle mounting the strongest responses which will be removed from the national herd. Over the years, our eradication efforts may just select for a BTB-susceptible population. The optimum test strategy is unlikely to be one where as many tests as logistically possible are conducted.

In conclusion, screening-test performance may be acceptable but up-to-date test specificity estimates should be quantified as a priority and analyses of test-contributions to herd ‘breakdown’ prevalence conducted. Test protocols applied once BTB is confirmed in one or more test-positive animals are likely to be suboptimal. The frequent application of less specific tests is likely to contribute substantially to the perceived UK BTB problem, triggering ever more intensive testing while removing large numbers of healthy animals from the national herd. Rather than focussed on test sensitivity and identifying reactors alone, the optimum test strategy will be risk-based and tailored to specific field situations. Simple adjustments may provide important improvements. First, for each herd test, the probability density of number of expected false positives should be drawn and, if a breakdown is not confirmed, inform whether subsequent testing is indicated. If the number of positives is within what is to be expected of the test and BTB presence cannot be confirmed then there is no sound reason to believe that *M. bovis* must be present in the herd. Second, if a herd breakdown is confirmed, confidence limits should be calculated for the initial test-derived estimate of the herd prevalence and this should inform the subsequent test protocol. Importantly, tests sacrificing specificity for sensitivity should only be used in high-prevalence herds. Multiplex serology tests, apparently more sensitive than γIFN at higher specificities, have become available in recent years [58], [59]. Third, by altering cut-offs, sensitivity and specificity of the available tests could be adjusted to suit specific herd test situations. For example, SICCT sensitivity could be increased for pre-movement testing. Fourth, after the risks densities leaving infected animals in the herd and removing non-infected animals have been drawn, a stochastic cost-benefit analysis should determine whether a herd test is indeed desirable. Similarly, cost-benefit analyses, co-informed by estimated BTB transmission rates, should inform the optimum test intervals. While providing increased value for money for the tax payer, it is likely that such exercises would limit the loss of healthy cows as well as distress to many farmers and the spread of disease between farms.
